# Clear Skies, Cloudy Mind: Probiotic-Related Brain Fogginess in a Commercial Airline Pilot

**DOI:** 10.7759/cureus.66426

**Published:** 2024-08-08

**Authors:** Piercarlo Minoretti

**Affiliations:** 1 Occupational Health, Studio Minoretti, Oggiono, ITA

**Keywords:** occupational health, rifaximin, probiotics, airline pilot, brain fog

## Abstract

Probiotics are widely consumed for their potential health benefits, particularly in promoting gastrointestinal health and treating functional gastrointestinal disorders (FGIDs). However, recent studies have raised concerns about the potential association between probiotic use and brain fog, a cognitive dysfunction characterized by confusion, impaired judgment, and lack of focus. A 47-year-old male commercial airline captain with over 10000 flight hours presented with a two-month history of bloating, abdominal distension, and irregular bowel habits following a period of occupational stress and irregular dietary habits. The pilot's previous medical history was largely uneventful, with the exception of a long-standing gastritis diagnosis. To manage this condition, he had been on a daily regimen of 20 mg of pantoprazole for approximately eight years. After a telemedicine consultation, he began taking an over-the-counter probiotic supplement containing 16 strains. Within five days, he experienced a significant exacerbation of abdominal symptoms, accompanied by somnolence, difficulty concentrating, and mental fatigue, raising safety concerns given his profession. Functional gastrointestinal examination revealed a distended abdomen with increased bowel sounds. Probiotic-associated brain fogginess was suspected, and the patient was advised to discontinue the supplements. Rifaximin therapy was initiated, resulting in rapid resolution of both gastrointestinal and cognitive symptoms. The clear temporal association between probiotic intake and symptom onset, followed by resolution after antibiotic treatment, suggests a causal relationship. This case highlights the potential risks of unsupervised probiotic use, particularly in safety-sensitive professions such as commercial aviation. Occupational health physicians and aeromedical examiners should be aware of the potential for probiotic-induced brain fog in airline pilots (APs). Prompt recognition and appropriate antibiotic treatment can result in complete symptom resolution and prevent occupational hazards.

## Introduction

Digestive symptoms are prevalent among commercial airline pilots (APs) due to their demanding and irregular work schedules, which can lead to disrupted meal times, unbalanced and irregular diets, depressive symptoms, altered circadian rhythms, and poor sleep quality [1]. In a study conducted by Lindgren et al., involving 354 pilots on duty in a Swedish airline company, the authors found that 62.1% of the participants experienced bloating, 15.2% suffered from heartburn, 14.4% reported epigastralgia, 12.4% had diarrhea, 9.9% experienced poor appetite, and 9.3% dealt with constipation [2]. The authors also revealed a strong correlation between insomnia and digestive symptoms among pilots, indicating that stress may play a significant role in the development of digestive issues within this occupational group [2]. Similarly, in a cross-sectional study by Li et al., which included 212 male pilots working for a Chinese civil airline company, functional gastrointestinal disorders (FGIDs) were reported in 83 (39.22%) of the study participants [3]. Interestingly, the authors found that flight level, high-salt food consumption, and sleep performance were independently associated with the occurrence of FGIDs [3]. In a prior seroprevalence investigation conducted on a cohort of 100 APs, we identified a notable prevalence (38%) of *Helicobacter pylori* seropositivity within this occupational group [4]. Furthermore, individuals in this professional category exhibited a 2.30-fold increased likelihood of *H. pylori* seropositivity when compared to a control group of office workers [4]. In a separate investigation, we have also highlighted a notable reduction in health-promoting bacterial species within the gut microbiota of APs [5]. The elevated *H*. pylori infection rate, combined with intestinal dysbiosis, could potentially contribute to the reported high incidence of FGIDs within this occupational group [2-5].

In recent years, there has been a substantial surge in the worldwide use of over-the-counter probiotics, primarily driven by their perceived benefits in promoting gastrointestinal well-being and alleviating FGIDs [6]. While probiotic use has gained widespread popularity among the general public, with some strains receiving "Generally Recognized as Safe" status from regulatory bodies, it is crucial to acknowledge that the use of probiotic strains and formulations is not without potential risks [7,8]. Of particular concern is the association between certain probiotic strains, such as *Lactobacillus* species, and the production of bacterial metabolic byproducts (e.g., D-lactate and histamine) implicated in the pathogenesis of brain fogginess, a term describing a state of cognitive dysfunction characterized by symptoms including confusion, impaired judgment, and lack of focus [9-13]. This study presents a case of brain fogginess in a commercial AP, which rapidly developed following the ingestion of a complex probiotic supplement. While the symptoms quickly subsided following brief antibiotic treatment with rifaximin, this occurrence highlights the possible risks of using probiotics without medical supervision, especially in occupations where safety is paramount, such as in the field of commercial flight operations.

## Case presentation

A 47-year-old Italian male commercial airline captain with over 10000 flight hours presented with a two-month history of bloating, abdominal distension, and irregular bowel habits. The patient reported that these symptoms emerged following a period of intense occupational stress accompanied by irregular dietary habits. His past medical history was unremarkable, except for a previous diagnosis of gastritis, for which he had been prescribed a daily regimen of 20 mg pantoprazole for approximately eight years. The patient's medical history was devoid of any surgical procedures. Furthermore, he reported no history of depressive symptoms or chronic sleep disorders. From an occupational perspective, the patient's typical flight routes were domestic within Europe, with durations less than four hours and rest periods often shorter than one hour between flights. Due to the constraints of his irregular work schedule, the patient initially opted for a telemedicine consultation with his primary care physician to seek guidance on managing his gastrointestinal symptoms. During this virtual encounter, the physician provided preliminary recommendations, advising the patient to limit his carbohydrate consumption and incorporate probiotics into his diet. A follow-up in-person evaluation was scheduled for three weeks later to reassess his condition and refine his treatment plan as needed. In the interim, the patient elected to purchase from an online retailer an over-the-counter probiotic supplement, specifically marketed for gastrointestinal health, which contained a blend of 16 probiotic strains (Table 1).

**Table 1:** Probiotic strains contained in the over-the-counter supplement Each row represents a different probiotic strain, numbered from 1 to 16.

Strain number	Probiotic strain
1.	*Lactobacillus acidophilus*
2.	*Lactobacillus casei*
3.	*Lactobacillus plantarum*
4.	*Lactobacillus paracasei*
5.	*Lactobacillus salivarius*
6.	*Lactobacillus rhamnosus*
7.	*Lactobacillus bulgaricus*
8.	*Bifidobacterium lactis*
9.	*Bifidobacterium bifidum*
10.	*Bifidobacterium longum*
11.	*Bifidobacterium breve*
12.	*Lactococcus lactis*
13.	*Streptococcus thermophilus*
14.	*Bacillus subtilis*
15.	*Bacillus coagulans*
16.	*Saccharomyces boulardii*

However, following five days of probiotic supplementation, despite adhering to the recommended carbohydrate restriction, the patient experienced a marked worsening of his gastrointestinal symptoms, characterized by severe abdominal bloating and flatulence. Concurrently, he reported somnolence, difficulty concentrating, and mental fatigue. Notably, these cognitive symptoms were also experienced during flight operations, raising significant safety concerns. As a result, the patient promptly contacted his occupational health physician and scheduled an urgent appointment to address these issues and mitigate any potential risks to his ability to perform his duties safely. Upon physical examination, the patient presented with a tractable, distended abdomen accompanied by increased bowel sounds. All other physical findings were unremarkable. Given the clear temporal association between the exacerbation of bloating, the onset of cognitive symptoms, and the initiation of probiotics, a diagnosis of probiotic-associated brain fogginess was strongly suspected. In light of the safety-sensitive nature of the patient's profession as a pilot and his concerns about impaired focus, he was advised to immediately discontinue the probiotic supplements to mitigate any potential risks to his ability to operate an aircraft safely. Subsequently, rifaximin therapy was initiated at a dose of 400 mg twice daily for a period of one week, which resulted in a rapid resolution of both his gastrointestinal and cognitive symptoms.

## Discussion

While probiotics are, by definition, intended to provide beneficial effects on human health, several potential safety issues associated with their use have emerged in recent years [6-8]. These concerns include but are not limited to, the potential transfer of antibiotic-resistance genes to the resident gut microbiome, the induction of D-lactic acid, and bacterial overgrowth [8]. Although still a subject of debate, the potential for probiotics to induce brain fog is currently under investigation [9,10,14,15]. Our case report of brain fog associated with unsupervised probiotic consumption in an AP underscores the crucial importance of exercising caution when administering probiotic supplements, particularly for individuals employed in safety-critical occupations. In such professions, even minor reductions in mental acuity, cognitive function, concentration, and multitasking abilities could lead to severe repercussions.

In a landmark study, Rao et al. identified a set of symptoms including abdominal bloating, distension, gas, and brain fog, which were associated with the use of probiotics and potentially caused by small intestinal bacterial overgrowth (SIBO) [9]. The authors proposed that excessive D-lactic acid production by certain probiotic strains, particularly lactobacilli, can lead to D-lactic acidosis, a condition associated with cognitive symptoms such as confusion and difficulty concentrating [9]. Another potential mechanism may involve histamine production by specific probiotic strains, which can contribute to various symptoms, including cognitive disturbances, by stimulating microglia activation and causing focal brain inflammation [16-18]. Although the potential association between probiotic intake and the onset of brain fog in susceptible individuals has been challenged, our case supports this relationship [14]. In our patient, a clear temporal association was observed between the initiation of probiotic supplementation and the onset of brain fogginess. Moreover, the resolution of symptoms following antibiotic therapy suggests a causal relationship in the AP described in our report. We speculate that the mechanism may involve overgrowth of the probiotic bacteria, potentially leading to excessive production of proinflammatory or neurotoxic molecules. Unfortunately, routine clinical laboratory tests for measuring serum D-lactic acid or histamine are not readily available in the clinical laboratory setting, making it challenging to definitively identify the specific compound responsible for the pilot's symptoms. However, histamine remains a candidate, as some of the probiotic strains (*L. casei *and *L. bulgaricus*) contained in the supplement taken by the patient have been reported to potentially have histamine-producing capacity [19,20].

**Figure 1:**
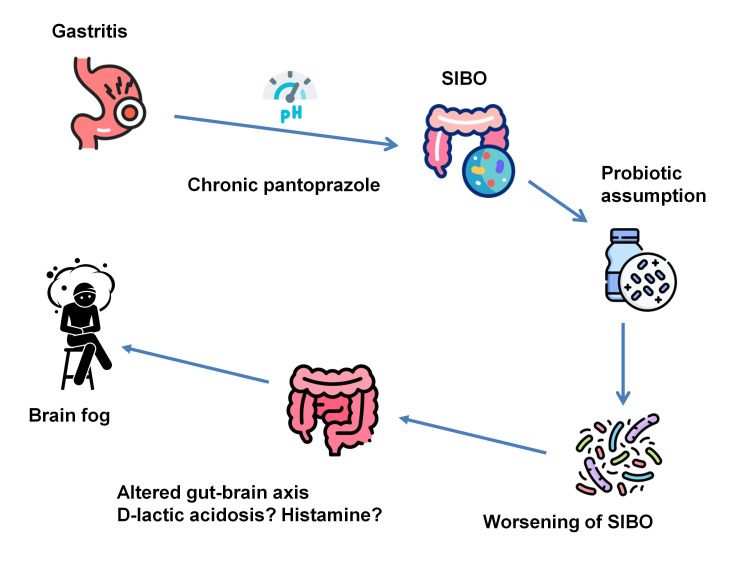
Schematic representation of the proposed pathophysiological cascade leading to brain fog in the described case The patient's long-term use of pantoprazole for approximately eight years, prescribed to manage his gastritis, may have led to an alteration in gastric pH. This change in the stomach's acidic environment could have facilitated the development of SIBO by creating conditions more favorable for bacterial proliferation. The subsequent introduction of probiotics exacerbated the microbial imbalance, ultimately worsening SIBO. This, in turn, resulted in altered gut-brain axis signaling, possibly mediated by D-lactic acidosis or histamine production. The culmination of these factors manifested in brain fog. SIBO: small intestinal bacterial overgrowth Image credits: Piercarlo Minoretti

In our patient, the constellation of symptoms (i.e., pre-existing bloating), exacerbation following probiotic supplementation, and subsequent amelioration with rifaximin therapy strongly suggested the presence of SIBO [9]. The exogenous probiotics likely exacerbated the overgrowth of resident bacteria. Notably, the patient's long-term use of a proton pump inhibitor may have facilitated SIBO development by suppressing gastric acid secretion, thereby creating a favorable environment for bacterial proliferation [21]. Alterations in gut microbiota composition and metabolic activity, as observed in SIBO and potentially precipitated by the intake of probiotics in susceptible individuals, may influence the gut-brain axis and subsequently affect cognitive function [22]. The hypothesized sequence of pathophysiological events culminating in brain fog for the patient under discussion is illustrated schematically in Figure 1.

This case report has several limitations, which should be considered within the context of a real-life occupational health setting. The absence of specific laboratory tests to measure D-lactic acid or histamine levels limits our ability to definitively identify the pathophysiological mechanism behind the patient's brain fog. Additionally, we did not conduct microbiome analysis before and after probiotic use, which could have provided valuable insights into changes in gut bacterial composition. Finally, we fully acknowledge that a formal diagnosis using conventional gastroenterological investigations was not obtained. However, given the safety-critical nature of the pilot's profession and the need for swift intervention, our approach prioritized symptom resolution and risk mitigation. We believe this case highlights the unique challenges faced in aviation medicine, where the balance between thorough diagnostic workup and ensuring immediate flight safety must be carefully managed. 

## Conclusions

Occupational health physicians and aeromedical examiners should be aware of the potential for probiotic-induced brain fog in APs, even in the absence of a known history of intestinal pathology. Obtaining a comprehensive history of probiotic supplement use is crucial in pilots who report the sudden onset of brain fog. Prompt recognition and appropriate antibiotic treatment can result in complete symptom resolution and prevent occupational hazards. Future studies should investigate the potential long-term effects of probiotic supplementation on cognitive function in commercial APs, a safety-sensitive occupation. Longitudinal studies comparing the incidence of brain fog between pilots who consume probiotics and those who do not could help establish clearer guidelines for probiotic use in this population. Additionally, further research on the gut-brain axis in pilots, considering their unique occupational stressors and irregular schedules, may also help elucidate the relationship between gastrointestinal health and cognitive function in this professional group.
